# Dairies Old and New

**Published:** 1887-12-03

**Authors:** 


### Dairies Old and New.

Few prettier sights could be witnessed in town or country fifty years ago than the rosy milkmaid, with her clean print frock and well-polished tin pail, starting out at six o'clock on a July morning for the rich meadows lying along the banks of the streams, where the deep-uddered cows awaited her coming. Town folk will no doubt think that a certain halo of romance surrounds the picture, and that if sober fact be desired, a grain of salt will not be out of place. But, indeed, the sober fact is that milkmaids in the olden time were not only proverbially, but really clean and neatly-dressed women, and if any servant were slatternly, she was never promoted to the dairy. The most elementary principle of dairy management is a scrupulous cleanliness. If you ask Mr. Shinyface, the country shopkeeper, why it is that Mrs. Bustler's butter is always worth a penny or three halfpence a pound more than Mrs. Lackaday's, he will tell you that Mrs. Bustler is the pink of cleanliness in her dairy: that from morning till night she keeps her maids constantly washing, scalding, scouring, and cleaning until you could take your dinner off the floor.

Dairies in the country are to some extent going out of fashion, and the milk and cream, with the butter-making and other processes, are being transferred to large towns. This remark does not so much apply to cheese dairies, or to those farms which are not within easy distance of great centres of population. But near London, Manchester, Glasgow, and similar places, it is found more convenient and profitable to send the first produce of the cow to a central dairy, placed in the midst of the consumers. One of the largest and best conducted of these central dairies is that of Messrs. Welford and Sons (Limited), at Elgin Avenue, Maida Vale. The quantity of milk received there daily from farms in the country is enormous, giving employment to upwards of a hundred persons in the daily distribution. The transference of such large quantities from the country cow-shed to the consumers in the metropolis is a business requiring the nicest management and the most scrupulous care. Milk, like fish and some kinds of fruit, requires to be used when it is fresh. After being drawn from the cow it begins to decompose very soon, and in the hot weather fermentation is especially rapid. Great precautions have, therefore, to be taken to ensure not only its prompt delivery to the customer, but also the most careful exclusion of everything of the nature of dirt or germs. Messrs. Welford's dairy is placed under the supervision of medical men specially certificated in sanitary science. It is the duty of those gentlemen not only to see that the central dairy is kept in the highest sanitary condition, but also from time to time to visit the sources of supply in the country, and to report upon them, as well as to visit specially any place which has become "suspect" for any reason. Special printed rules are issued to all farmers supplying milk, and in every cow-shed other printed rules are furnished to the men who have charge of the cows. If illness of any kind break out among the cattle the particular animal attacked is isolated until the nature of the illness is determined. In like manner, if any of the men in charge, or any members of their families, show signs of ill health of ever so slight a degree, the men are instructed to report the case at once, and to discontinue attendance at the farm until the disease is recovered from or declared to be non-infectious. In order to remove every possible temptation from the men to return too early to their duties, their wages are paid until the doctor pronounces the danger of infection to be past.

It might be supposed that these precautions merely exist on paper, and are not really carried out. That, however, would be a mistake. Self-interest alone, if no other reason, compels the proprietors of large dairies to do their utmost to avoid all kinds of infection in connexion with the milk supply. An outbreak of fever traced to such supply with certainty, or even with some degree of probability, might ruin a lucrative business in a few weeks. It is clearly therefore the interest, as well as the duty, of proprietors not only to have a strict code of regulations for every department of collection and distribution, but also to ensure, by personal supervision, the constant compliance of all their subordinates with such regulations. As human nature is constituted, the surest guarantee of good service is generally the certainty that the service rendered to us is also a service to the renderer. That may appear a little cynical, nevertheless the fact that the due supply of absolutely pure milk is profitable to the supplier as well as to the supplied will be felt to be an assurance upon which most men will place a good deal of reliance. Those who are interested in the question of the daily feeding of the enormous population of London will find much to instruct and gratify them if they will pay a visit to Messrs. Welford's dairy on their own account.

